# Notes and News

**Published:** 1903-01-17

**Authors:** 


### NOTES AND NEWS.

IT is stated that France is about to establish a bacteriological institute at Bangkok.

THE University of London is about to establish a Board of Studies in Human Anatomy and Morphology.

A POST-GRADUATE School of Hygiene and Medical Jurisprudence is to be established in connection with the University of Turin.

MR. G. CROMPTON and Mr. C. Crompton have endowed a bed in the Royal Infirmary, Derby, as a memorial to their brother, Mr. E. Crompton.

A GRAND evening concert will shortly be held at the Bechstein Hall in aid of the Royal Free Hospital. The concert is arranged under the auspices of the Ladies' Association.

IT appears probable that there will be another Egyptian Medical Congress in about five years' time. The Congress held in December was a success, but the British attendance was small.

TWO sanatoria for the insane who are consumptive have been opened by Glasgow District Lunacy Board. One is in connection with the Asylum at Gartloch, and the other with the Woodlee Asylum. The sanatoria have been constructed of iron and wood, at a cost of about £90 per bed. A patent system of air-spaced walls has been introduced by the contractors, Messrs. Spiers and Co, of Glasgow, and accommodation is provided for 80 patients.

DR. KOCH has started for Rhodesia, on the Commission of the Chartered Company, to study the prevalent cattle diseases. It is stated that he hopes one result will be that he will be able to substantiate his contention that bovine tuberculosis is not transmittable to man. Of the success he will have in combating the cattle disease he does not appear to be so sanguine. He is accompanied by Doctors Neufeld and Klein.

AMONGST the movements at the University of Edinburgh is one for the formation of a class for instruction in the administration of anæsthetics.

THE necessity for systematic vaccination is becoming very generally felt. Belgium now is about to discuss the question of compulsory vaccination and re-vaccination.

A NEW operating theatre, nurses' home, and dispensary have been opened by the Lord Lieutenant at the Adelaide Hospital, Dublin. The operating theatre cost £3,500. The Queen has permitted the nurses' home to bear her name.

AN international manifesto against the use of alcohol has been signed by about 300 British doctors and a smaller number of medical men of other nationalities, one Frenchman only being amongst the number. The document declares alcohol to be poison, not food.

THE hon. secretaries of the League of Mercy, which was founded by Royal Charter to promote the welfare of hospitals by securing support for King Edward's Hospital Fund, have received the following letter from Lieut.-Colonel Sir Arthur Bigge, G.C.V.O., etc., etc., Private Secretary to his Royal Highness the Prince of Wales, Grand President of the League of Mercy.

"York House, St. James's Palace, S.W.
January 6th, 1903.

DEAR SIR,—His Royal Highness, the President of King Edward's Hospital Fund, directs me to convey to the officials and members of the League of Mercy the expression of his thanks for the sum of £8,000 which has been handed over to the Fund by the League. This substantial contribution is an increase of £1,000 on the amount received last year, which his Royal Highness feels sure will be generally regarded as a practical proof of the excellent results accomplished by the earnest untiring work of the League of Mercy.

Yours very faithfully,
(Signed) ARTHUR BIGGE."

PROFESSOR LORENZ, of Vienna, gave a demonstration of his method of Reduction of Congenital Displacement of the Hips at the City Orthopædic Hospital, Hatton Garden, in the presence of members of the medical profession invited by the medical staff of the hospital. In the evening Professor Lorenz was entertained at dinner by Dr. Noble Smith, to which a large number of medical men were invited. Professor Lorenz gave a demonstration of his methods in Liverpool at an earlier date.

A NOVELTY in the way of charitable entertainments is announced for February 10th in the form of an Advertisement and Poudré Ball, at the Empress Rooms, in aid of the Metropolitan Hospital, Kingsland Road, which is greatly in need of funds. The ball is being organised by the Ladies' Association, and tickets with full particulars can be obtained from Mrs. J. Coope, Chairman of the Association, or from Mrs. Elphinstone-Maitland, Royal Palace Hotel. There is a long and influential list of patrons, and the guinea tickets include supper.

